# GmMYB176 Regulates Multiple Steps in Isoflavonoid Biosynthesis in Soybean

**DOI:** 10.3389/fpls.2019.00562

**Published:** 2019-05-03

**Authors:** Arun Kumaran Anguraj Vadivel, Justin Renaud, Sateesh Kagale, Sangeeta Dhaubhadel

**Affiliations:** ^1^London Research and Development Centre, Agriculture and Agri-Food Canada, London, ON, Canada; ^2^Department of Biology, Western University, London, ON, Canada; ^3^National Research Council Canada, Saskatoon, SK, Canada

**Keywords:** soybean, chalcone synthase, MYB transcription factor, isoflavonoids, gene regulation, gene expression, specialized metabolism

## Abstract

Isoflavonoids are a group of plant natural compounds synthesized almost exclusively by legumes, and are abundant in soybean seeds and roots. They play important roles in plant-microbial interactions and the induction of nod gene expression in Rhizobia that form nitrogen-fixing nodules on soybean roots. Isoflavonoids also contribute to the positive health effects associated with soybean consumption by humans and animals. An R1 MYB transcription factor GmMYB176 regulates isoflavonoid biosynthesis by activating *chalcone synthase* (*CHS*) 8 gene expression in soybean. Using a combination of transcriptomic and metabolomic analyses of GmMYB176-RNAi silenced (GmMYB176-Si), GmMYB176-overexpressed (GmMYB176-OE), and control soybean hairy roots, we identified a total of 33 differentially expressed genes (DEGs) and 995 differentially produced metabolite features (DPMF) in GmMYB176-Si hairy roots, and 5727 DEGs and 149 DPMFs in GmMYB176-OE hairy roots. By a targeted approach, 25 isoflavonoid biosynthetic genes and 6 metabolites were identified as differentially regulated in GmMYB176-OE and GmMYB176-Si soybean hairy roots. Taken together, our results demonstrate the complexity of isoflavonoid biosynthesis in soybean roots and suggest that a coordinated expression of pathway genes, substrate flux and product threshold level may contribute to the dynamic of the pathway regulation.

## Introduction

**FIGURE 1:**
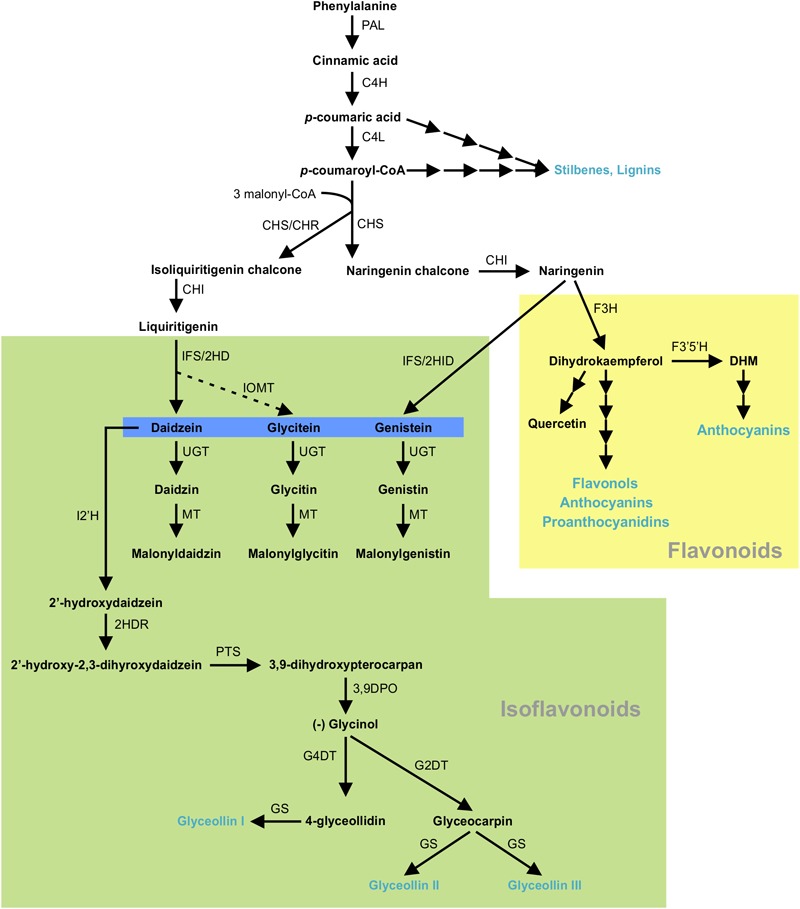
(Iso)flavonoid biosynthesis pathway in soybean. The multiple arrows indicate multiple steps in the pathway and the dotted arrow indicates speculated steps. PAL, phenylalanine ammonia lyase; C4H, cinnamate 4-hydroxylate; 4CL, 4-coumarate-CoA ligase; CHS, chalcone synthase; CHR, chalcone reductase; CHI, chalcone isomerase; IFS, 2-hydroxyisoflavanone synthase; 2HID, 2-hydroxyisoflavanone dehydratase;IOMT, isoflavone O-methyltransferase; UGT, uridine diphosphate glycosyltransferase; MT, malonyltransferase; I2′H, Isoflavone 2′-hydroxylase; 2HDR, 2′-hydroxydaidzein reductase; F3H, flavanone-3-hydroxylase; F3′5 ′H, flavonoid 3′5′-hydroxylase; DHM, dihydromyricetin; PTS, pterocarpan synthase; 3,9 DPO, 3,9-dihydroxypterocarpan 6a-monooxygenase; G4DT, glycinol 4-dimethylallyltransferase; G2DT, glycinol 2- dimethylallyltransferase; GS, glyceollin synthase. Flavonoid and isoflavoboid branches are highlighted by yellow and green, respectively. Isoflavone aglycones are highlighted in blue. Blue texts indicate the end products of that branch.

Isoflavonoids are specialized metabolites of dual importance for plant-environment interactions. They act as signaling molecules for symbiosis during nitrogen fixation with *Rhizobia* (Phillips and Kapulnik, 1995; Ferguson and Mathesius, 2003; Subramanian et al., 2006) and as phytoalexins with antimicrobial properties (Lozovaya et al., 2004a; Lygin et al., 2013). Isoflavonoids are synthesized through a legume-specific branch of phenylpropanoid pathway that is regulated by a coordinated expression of several structural genes. As shown in Figure 1, conversion of phenylalanine to cinnamic acid by phenylalanine ammonia lyase (PAL) initiates the general phenylpropanoid biosynthetic pathway leading into the production of a plethora of biologically active compounds. Some of the early downstream enzymes of the phenylpropanoid pathway, after PAL, are cinnamate 4-hydroxylase (C4H), 4 coumoryl-CoA ligase (4CL) and chalcone synthase (CHS), with the latter being a key step in directing C6–C3 carbon skeletons into the flavonoid/isoflavonoid branch of the phenylpropanoid pathway. CHS converts *p*-coumaroyl CoA and three molecules of malonyl CoA to naringenin chalcone or it coacts with chalcone reductase (CHR) to produce isoliquiritigenin chalcone, thereby channeling the metabolic flux into iso(flavonoid) biosynthesis (Figure 1). Previously, we showed that *GmCHS7* and *GmCHS8* genes play a critical role in isoflavonoid biosynthesis in soybean (*Glycine max*) seeds (Dhaubhadel et al., 2007). Soybean is a paleopolyploid with most genes present as a member of large gene families. Studying expression levels of members of a gene family can be challenging due to their high sequence identity (Blanc and Wolfe, 2004; Shoemaker et al., 2006; Panchy et al., 2016).

The majority of phenylpropanoid biosynthetic genes are regulated by MYB transcription factors (Jin and Martin, 1999; Boddu et al., 2006; Albert et al., 2014; Lin-Wang et al., 2014). The expression of maize *PAL, CHS, flavanone 3-hydroxylase* (*F3H*) and *dihydroflavonol 4-reductase* (*DFR*) genes involved in anthocyanin biosynthesis are coordinately regulated by a bHLH protein-encoding gene R and a MYB gene C1 (Grotewold et al., 1998; Bruce et al., 2000; Kong et al., 2012). Heterologous expression of the maize C1 and R transcription factors in soybean increased total isoflavone levels (Yu et al., 2003). Recently, a coordinated expression of pathway genes was demonstrated for maize phenylpropanoids where a set of 11 transcription factors recognize 10 or more promoters (Yang et al., 2017). Knowledge of such an organized gene regulatory network will be useful for manipulating the pathway in metabolic engineering efforts.

In soybean, several MYB transcription factors have been reported to regulate the key isoflavonoid biosynthetic genes and affect isoflavonoid accumulation. Overexpression of *GmMYB29* (Chu et al., 2017), *GmMYB133* (Bian et al., 2018), *GmMYB12B2* (Li et al., 2013), *GmMYB58* and *GmMYB205* (Han et al., 2017) play a positive role in the regulation of isoflavonoid biosynthesis while *GmMYB100* (Yan et al., 2015) and *GmMYB39* (Liu et al., 2013) inhibit isoflavonoid accumulation by suppressing the expression of multiple isoflavonoid genes. Previously we showed that down regulation of *GmCHS8* expression reduced isoflavonoid levels in soybean hairy roots (Yi et al., 2010). This was achieved by RNAi silencing of *GmMYB176* (Glyma.05G032200) in soybean and monitoring its effect on *GmCHS8* expression and metabolite accumulation. GmMYB176 may regulate multiple genes in the isoflavonoid biosynthetic pathway, which may, in turn, affect the production of metabolites upstream or downstream of isoflavone aglycones.

Since gene regulation is a dynamic process and changes in transcript level may not necessarily correlate with the protein level or the metabolite level, here we aim to portray all possible connections between GmMYB176 and its potential target genes, and resulting metabolites in soybean. Soybean hairy root lines with *GmMYB176* silenced (GmMYB176-Si) and *GmMYB176* overexpressed (GmMYB176-OE) were developed, and their transcriptome and metabolome were compared with wild type hairy roots (control). We identified a total of 33 differentially expressed (DE) genes and 995 differentially produced metabolites in GmMYB176-Si hairy roots, and 5727 DE genes and 149 differentially produced metabolites in GmMYB176-OE hairy roots compared to the control. A targeted analysis of the isoflavonoid pathway identified 25 isoflavonoid biosynthetic genes and 6 metabolites as differentially regulated in GmMYB176-OE and GmMYB176-Si soybean hairy roots compared to control roots. The integration of these two approaches led to a better understanding of isoflavonoid biosynthesis in soybean.

## Materials and Methods

### Plasmid Construction

The coding region of *GmMYB176* was amplified from soybean root cDNA and recombined into the Gateway destination vectors, pK7GWIWG2D (II) and pK7WG2D (Karimi et al., 2002), to obtain silencing (pGmMYB176-Si) and overexpression (pGmMYB176-OE) constructs, respectively. The recombinant plasmids were then transformed into *Agrobacterium rhizogenes* K599 by electroporation.

### Plant Material, Growth Condition, and Generation of Soybean Hairy Roots

To obtain soybean cotyledons for hairy root transformation, soybean (*Glycine max* L. Merr.) cv Harosoy63 seeds were grown as described by Dastmalchi et al. (2016). Six-day-old soybean cotyledons were harvested and transformed with *A. rhizogenes* K599 carrying silencing (pGmMYB176-Si) or overexpressing (pGmMYB176-OE) constructs, according to the method from Subramanian et al. (2005). Transgenic hairy roots were selected using a GFP marker under a Leica MZ FL III fluorescence stereomicroscope with a GFP filter. Control soybean hairy roots were also generated using *A. rhizogenes* K599 only. The root samples were flash frozen, and stored at -80°C until use.

### RNA Extraction, Transcriptome Sequencing and Read Pre-processing

RNA was extracted from four pooled replicates (100 mg each) of GmMYB176-OE, GmMYB176-Si and control soybean hairy root samples using RNeasy plant mini kit (Qiagen, United States). The RNA quality and concentration were checked in Agilent Bioanalyzer (Agilent, United States). TruSeq RNA sequencing libraries were constructed following the standard preparation guide (Illumina, United States). All 12 samples were multiplexed in a lane of a flow cell, and paired-end sequencing was completed using the Illumina HiSeq 2500 platform at the National Research Council of Canada (Saskatoon, Canada). Before read mapping and expression quantification, all reads were filtered using Trimmomatic version 0.36 (Bolger et al., 2014).

### Read Mapping, Differential Expression Analysis and Functional Annotation

Pair-end sequencing reads were aligned end-to-end to the *G. max* reference genome (Gmax_275_v2.0; softmasked sequence) with STARv2.5.2b (Dobin et al., 2013), allowing a maximum of two mismatches in an alignment. Read alignments were reported only if both reads in a pair were mapped and showed concordant results. Alignments that contained non-canonical junctions were filtered out. Gene-level raw read counts were obtained using the htseq-count tool from the HTSeq *python* library (Anders et al., 2015), using the “intersection_nonempty” mode and Gmax_275_wm82.a2.v1 gene_exon annotation. Raw gene counts are loaded and normalized using regularized log (rlog) transformation which minimizes differences between samples for rows with small counts, and normalizes with respect to library size^1^. Differential expression between controls and GmMYB176-OE or controls and GmMYB176-Si samples was evaluated using DESeq2 v1.14.0 (Love et al., 2014). GO annotation categories and pathway enrichment analyses were carried out using Glyma identifiers of DEGs in PhytoMine^2^. Data from KEGG and PlantCyc (Plant Metabolic Network (PMN)^3^) resources were used to conduct pathway enrichment analyses, with the *Glycine max* database selected as the reference. The Bonferroni statistical test was used to adjust the *p*-values for multiple hypotheses. RNAseq data are available in the ArrayExpress database under accession number E-MTAB-7379.

### Quantitative RT-PCR Analysis

The same RNA samples utilized for RNAseq library preparation were used for qRT-PCR analysis. Total RNA (1 μg) was reverse transcribed using the ThermoScript^TM^ RT-PCR System (Invitrogen, United States). Gene-specific primers sequences for qPCR are in listed in Supplementary Table S1. All reactions were performed in three technical replicates, and the expression was normalized to the reference gene *CONS4* (Libault et al., 2008). The data were analyzed using CFX manager (BioRad, United States).

### Metabolite Extraction

Frozen hairy root samples (60 mg) were ground with liquid nitrogen and extracted in 5 mL of methanol: 1.5 M hydrochloric acid (80:20, v/v). Since the predominant isoflavones in soybean hairy roots are their malonylated conjugates (Lozovaya et al., 2004b), this method led to the hydrolysis of malonylated isoflavones and allowed for the detection of both the glycosylated and aglycone isoflavones in the root samples. The samples were sonicated first at ambient temperature for 30 min, and then at 40°C for additional 30 min followed by incubation in a 100°C water bath for 30 min with intermittent vortexing. The extracts were cooled to room temperature, shaken at 300 rpm for 4 h and centrifuged at 1000 *g* for 20 min at ambient temperature. The supernatant was filtered through a 0.45 μm syringe filter (Millipore, United States), and then dried at 50°C under nitrogen gas for 30 min. The dried pellet was washed with 1 mL of ethyl acetate twice and then dried under nitrogen gas. The dried metabolite extracts were dissolved in 50% methanol and used in HPLC analysis. All samples were treated exactly the same, and non-targeted metabolomics comparisons were performed based on relative changes in metabolite abundance, measured by peak area. Any alteration to the types of isoflavone conjugates present is likely to be altered equivalently across the samples.

### LC-MS Data Acquisition and Analysis

A Q-Exactive Quadrupole Orbitrap mass spectrometer (Thermo Fisher Scientific, United States) coupled to an Agilent 1290 HPLC was used for high resolution LC-MS analysis. The HPLC system was equipped with a Zorbax Eclipse Plus RRHD C18 column (2.1 × 50 mm, 1.8 μm) maintained at 35°C. Samples (5 μL each) were run with a flow rate of 0.3 mL min^-1^. Water with 0.1% formic acid and acetonitrile with 0.1% formic acid was used as mobile phases A and B, respectively (Optima grade, Fisher Scientific, United States). Mobile phase B was held at 0% for 0.5 min and increased to 100% over 3.0 min. It was held at 100% for 2 min before returning to 0% over 30 s. Heated electrospray ionization (HESI) conditions used are as follows; spray voltage, 3.9 kV (HESI+), 3.7 kV (HESI-); capillary temperature, 400°C; probe heater temperature, 450°C; sheath gas, 17 arbitrary units; auxiliary gas, 8 arbitrary units; and *S*-Lens RF level, 45. Compounds were detected using 140,000 resolutions, full mass scans between the range of *m/z* 100 to 1200 in separate positive and negation ionization runs. Automatic gain control (AGC) target and maximum injection time (IT) were 3 × 10^6^ and 500 ms, respectively. Separate data-dependent acquisition (DDA) mode experiments were used for one sample for each treatment to facilitate compound identification. These DDA methods used identical HESI conditions and comprised of a full MS scan at 35,000 resolution between *m/z* range of 100 to 1200, AGC target of 3 × 10^6^ and maximum IT of 128 ms. The top 10 most intense ions above a threshold of 8 × 10^5^ were sequentially selected for MS/MS using a 1.0 *m/z* isolation window, normalized collision energy (NCE) of 30 and excluded from subsequent MS/MS for 5 s.

Compounds were identified using commercial standards when possible. Raw files were converted to mzml format using ProteoWizard (Kessner et al., 2008) with peak filter applied, and imported into R using the XCMS package (Smith et al., 2006). Features were detected with centWave (ppm tolerance of 1.0) (Tautenhahn et al., 2008). Prefilter was 6 scans with minimum 5000 intensity, signal to noise threshold was 5, and the noise was set to 3 × 10^6^ and 1 × 10^6^ for positive and negative modes, respectively. Retention time correction was applied using obiwarp (Prince and Marcotte, 2006). Features were present in at least 25% of all samples were grouped, *m/z* width set to 0.015 and retention time deviation was 10 s. Default settings on “fillPeaks” function were used and zeros imputed with two-thirds the minimum value on a per mass basis.

## Results

### Expression Analysis of *GmMYB176* in Transgenic Soybean Hairy Roots

**FIGURE 2:**
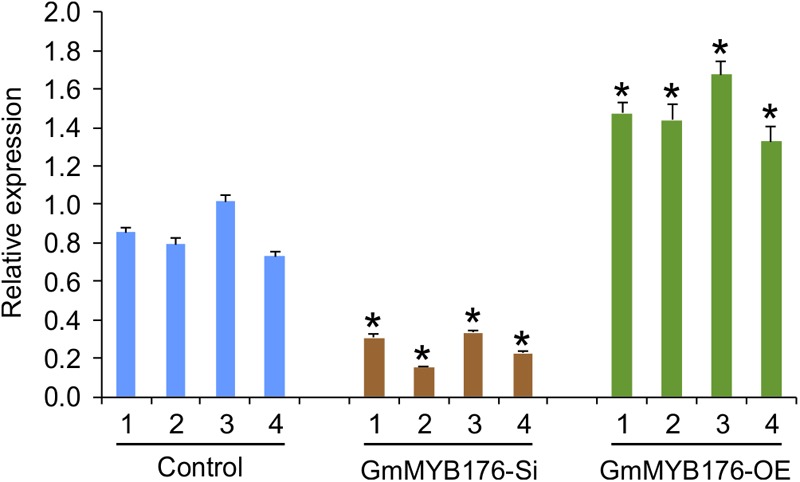
Expression levels of *GmMYB176* in the samples used for RNAseq analysis. Total RNA extracted from GmMYB176-Si, GmMYB176-OE, and control samples was used for qPCR analysis. Error bars indicate SEM. Values were normalized against the reference gene, *CONS4*. Asterisks represent the significant differences with the control samples at *p* < 0.05.

To investigate the global effect of GmMYB176 on gene expression and metabolite production, soybean hairy roots were generated where *GmMYB176* was either silenced (GmMYB176-Si) or overexpressed (GmMYB176-OE) using CaMV 35S constitutive promoter. Transgenic hairy roots co-expressed GFP as the plasmids contained a GFP marker as a separate built-in cassette. The roots with strong GFP expression were collected and pooled. Each transgenic hairy root is an independent transformation event where the pooled sample set represented a biological replicate. RNA was extracted from multiple biological replicates of GmMYB176-Si, GmMYB176-OE and control hairy roots, and the expression level of *GmMYB176* was measured. Even though variations in the level of *GmMYB176* transcript was observed within the sample sets, its accumulation was increased in GmMYB176-OE, and reduced in GmMYB176-Si samples compared to the controls (Supplementary Figure S1). Four representative samples from each category that showed greater difference in *GmMYB176* transcript level compared to control samples were selected from Supplementary Figure S1 for the global transcriptome analysis (Figure 2).

### Data Quality and Coverage of Soybean Transcriptome

**FIGURE 3:**
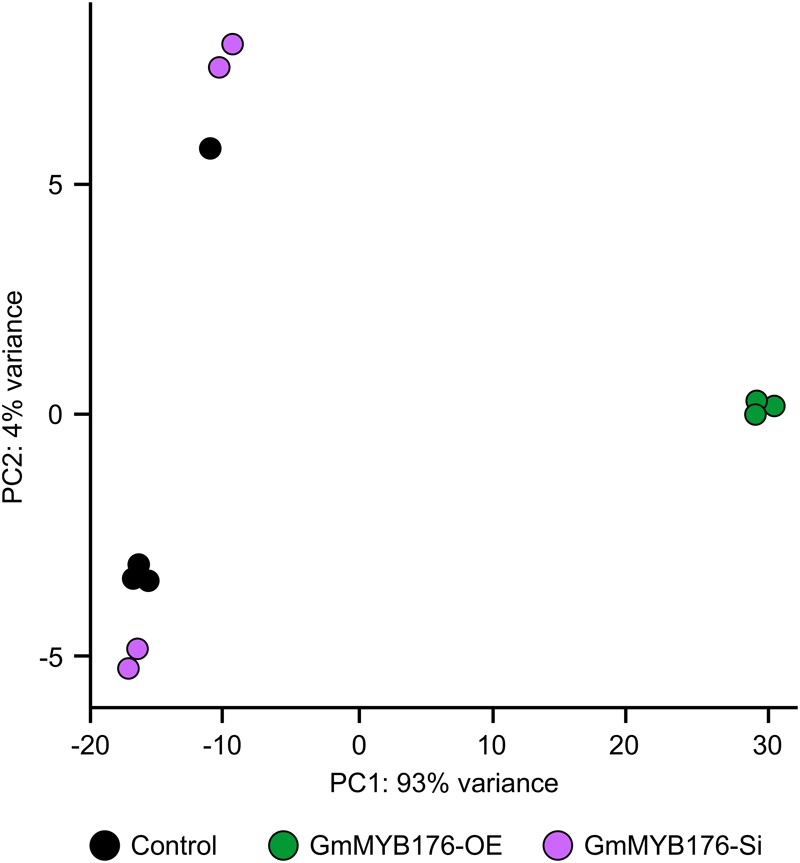
PCA plot for GmMYB176-Si, GmMYB176-OE and control hairy roots. PCA reveals a transcriptome shift when *GmMYB176* transcripts level was altered. Each dot represents one replicate and there were 4 biological replicates for each of the samples used for the RNA sequencing analyses.

The hairy root transcriptomes of GmMYB176-Si, GmMYB176-OE, and control soybean lines with four replicates each were sequenced using 100 bp paired-end reads. The resulting data were then mapped against the soybean transcriptome v2.0 (Wm82.a2.v1). The mapping and quality information including total reads per RNA sequencing library, mapped reads, uniquely mapped reads and gene coverage (%) are shown in Table 1. Coverage of the 56,044 protein-coding loci ranged from 74.74 to 77.04%. As indicated by the PCA plot, a large variation among the biological replicates, especially in GmMYB176-Si and control samples were observed, indicating the heterogeneity of biological samples. The results also revealed a clear transcriptome shift between control samples and GmMYB176-OE samples (Figure 3). Biological variation for all the gene models in three samples, GmMYB176-Si, GmMYB176-OE, and controls was displayed using dispersion graphs (Supplementary Figure S2). Dispersion plots shows the tight dispersion overall indicating good quality RNA sequencing libraries for the three sample types.

**Table 1:** RNA sequencing reads and coverage of soybean transcriptome.

Sample	Replicate	Total Reads	Mapped Reads	Uniquely Mapped	Gene Coverage (%)
Control	1	13389565	9727667	9602622	74.74
	2	22454365	17349519	17130185	76.02
	3	15761275	12261354	12112510	75.31
	4	18983435	14871395	14681029	76.76
GmMYB176-Si	1	13997019	11418283	11266836	74.74
	2	19032775	14305367	14107464	75.39
	3	17811575	14871462	14683603	76.43
	4	20314253	16269587	16058374	77.04
GmMYB176-OE	1	19556135	15581413	15371028	76.76
	2	15777590	12904965	12736516	76.22
	3	19070654	13694580	13503216	76.18
	4	15257415	10742872	10596737	75.49

### Differential Gene Expression Analysis and Their Functional and Structural Annotation

The differential gene expression analysis was carried out in R using DESeq2 v1.14.0. The Bonferroni adjusted *p*-value distribution for all genes assessed for differential expression between GmMYB176-OE and controls showed a large number of significantly differentially expressed genes with a uniform dispersion (Supplementary Figures S2, S3). Two lists were generated from pair wise differential expression analyses: (i) GmMYB176-Si with control and (ii) GmMYB176-OE with control. A total of 5727 genes were differentially expressed in GmMYB176-OE tissues compared to the control, whereas, only 33 genes were differentially expressed in GmMYB176-Si tissues compared to the control at two fold cut-off with *p* < 0.001. There were 3337 genes upregulated and 2390 genes downregulated in GmMYB176-OE tissues, whereas 26 genes upregulated and 7 genes downregulated in GmMYB176-Si tissues compared to the controls (Supplementary Table S2).

The “Glyma IDs” of DE genes were used for gene ontology (GO) analysis in PhytoMine^4^. DE genes in GmMYB176-OE tissues in comparison to controls were grouped into 17 GO “Biological process” categories, and 15 “Molecular function” categories (Supplementary Figure S4). DEGs identified in the GmMYB176-Si tissues relative to controls (Supplementary Table S2) did not group into any GO annotation category. In order to retrieve differentially expressed phenylpropanoid pathway genes in GmMYB176-Si and GmMYB176-OE samples compared to control, a pathway enrichment analysis was performed (Supplementary Figure S5). The results revealed that overexpression of GmMYB176 altered the expression of genes involved in several metabolic processes. All the DEGs identified under flavonoid biosynthesis were down regulated while 15 DEGs identified as isoflavonoid biosynthetic genes contained 9 down- and 6- upregulated genes. A detailed investigation of isoflavonoid related DEGs identified 14 of them as peroxidases. Therefore, a targeted approach was conducted to uncover isoflavonoid pathway-specific DEGs in GmMYB176-Si and GmMYB176-OE samples compared to controls.

### Differentially Expressed Phenylpropanoid Pathway Genes

Previously we demonstrated that GmMYB176 regulates *CHS8* gene expression in soybean and affects isoflavonoid biosynthesis (Yi et al., 2010). To determine if GmMYB176 co-regulates other genes in the isoflavonoid biosynthetic pathway, we searched for isoflavonoid genes in the list of DEGs. To do this, we first prepared a gene list that contained 94 putative and confirmed isoflavonoid biosynthetic genes belonging to 10 gene families (Supplementary Table S3), and searched each gene against the annotation list of 5727 DEGs in GmMYB176-OE and 33 DEGs in GmMYB176-Si (Supplementary Table S4). This search identified a total of 25 DEGs as isoflavonoid biosynthetic genes in GmMYB176-OE samples. As shown in Table 2, out of 7 *GmCHS* DEGs, transcripts of 3 *GmCHS* gene family members (*GmCHS1, GmCHS3a*, and *GmCHS9*) were 2.2 to 2.3 times higher in GmMYB176-OE tissues compared to control whereas transcripts of other 4 *GmCHS*s including *GmCHS6* (2.8 times), *GmCHS8* (2 times), *GmCHS10* (2.5 times), and *GmCHS11* (2.55 times) were lower in the same tissues compared to control demonstrating the differential regulation of members of the same gene family. Our results revealed that over expression of GmMYB176 affects almost the entire isoflavonoid pathway as transcripts for upstream enzymes such as PAL, C4H, GmCHI1B2, GmIFS1 and downstream enzymes such as prenyltransferases, P450s and reductases were down-regulated (Table 2). No difference in the expression of those genes was observed in GmMYB176-Si hairy roots compared to controls.

**Table 2:** Effect of overexpression of *GmMYB176* on the expression of isoflavonoid biosynthetic genes.

	Locus ID	FC	padj	Annotation
Downregulated				
	Glyma.02G309300	0.36	1.6E-20	*Phenylalanine ammonialyase*
	Glyma.10G058200	0.47	2.5E-63	*Phenylalanine ammonialyase*
	Glyma.20G180800	0.38	2.3E-33	*Phenylalanine ammonialyase*
	Glyma.20G114200	0.03	1.9E-26	*Cinnamate 4-hydroxylase (GmC4H)*
	Glyma.07G202300	0.35	4.0E-62	*Isoflavone synthase (GmIFS1)*
	Glyma.09G075200	0.31	1.3E-05	*Chalcone synthase (GmCHS6)*
	Glyma.11G011500	0.49	4.8E-20	*Chalcone synthase (GmCHS8)*
	Glyma.02G130400	0.35	6.2E-04	*Chalcone synthase (GmCHS10)*
	Glyma.01G091400	0.35	1.6E-04	*Chalcone synthase (GmCHS11)*
	Glyma.10G292200	0.27	3.3E-38	*Chalcone isomerase (GmCHI1B2)*
	Glyma.01G134600	0.18	2.7E-28	*Prenyltransferase (GmPT01)*
	Glyma.10G295300	0.31	4.1E-45	*Prenyltransferase (GmPT10a)*
	Glyma.10G070300	0.05	1.1E-08	*Prenyltransferase (GmPT10d)*
	Glyma.11G210300	0.05	4.4E-45	*Prenyltransferase (GmPT11a)*
	Glyma.20G245100	0.45	8.0E-11	*Prenyltransferase (GmPT20)*
	Glyma.15G156100	0.48	2.7E-04	*Isoflavone 2′-monooxygenase*
	Glyma.18G220500	0.46	2.9E-48	*Vestitone reductase*
	Glyma.09G269600	0.35	1.0E-31	*Vestitone reductase*
	Glyma.09G269500	0.47	2.7E-08	*Vestitone reductase*
Upregulated				
	Glyma.08G109400	2.30	2.1E-10	*Chalcone synthase (GmCHS1)*
	Glyma.08G109300	2.20	5.6E-05	*Chalcone synthase (GmCHS3a)*
	Glyma.08G109500	2.28	1.2E-29	*Chalcone synthase (GmCHS9)*
	Glyma.16G219500	3.52	1.6E-14	*Chalcone reductase (GmCHR16B)*
	Glyma.14G098100	3.13	2.8E-23	*Chalcone isomerase (GmCHI3C1)*
	Glyma.17G226600	2.18	6.7E-15	*Chalcone isomerase (GmCHI3C2)*

*FC, fold change.*

**FIGURE 4:**
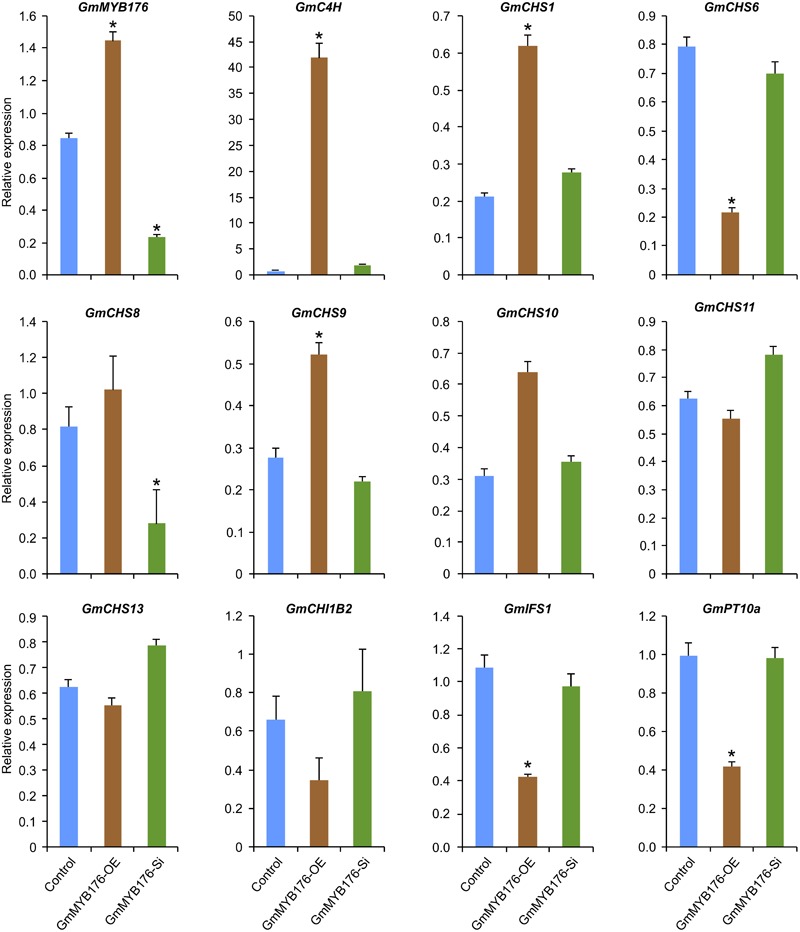
Expression analysis of candidate phenylpropanoid genes in GmMYB176-Si, GmMYB176-OE and control hairy roots. The RNA samples used in RNAseq library preparation were subjected to qPCR analysis of isoflavonoid genes using the gene-specific primers. Relative expression corresponds to mean gene expression in four biological replicates, with three technical triplicates. Error bars indicate SEM. Values were normalized against the reference gene *CONS4*. Asterisk indicates the significant difference in transcript level at *p* < 0.05 compared to control.

To validate the RNAseq results, selected genes from Table 2 were subjected to a qRT-PCR analysis. Same RNA samples that were utilized in the preparation of the RNAseq libraries were used for the qRT-PCR. Primer sequences were designed to amplify unique regions within the target genes to differentiate the targets from their gene family members. The amplicons for each target gene was sequenced to confirm the specificity. The transcript levels of *GmMYB176* in GmMYB176-Si and GmMYB176-OE samples were confirmed by qPCR earlier (Figure 2). As shown in Figure 4 and Table 2, the expression levels of *GmIFS1, GmCHS6*, and *GmPT10a* were consistent with the patterns apparent in our RNAseq analysis. However, RNAseq results for other genes in Table 2 did not correlate with the qPCR analysis. For example, our qPCR analysis displayed a 38.5 times higher expression of *GmC4H* in GmMYB176-OE tissues and no change in expression in GmMYB176-Si tissues compared to controls (Figure 4). In contrary, the RNAseq analysis indicated a 28.5 times reduced level of *GmC4H* in GmMYB176-OE tissues than in control (Table 2). Transcript abundance of *GmCHS8* in GmMYB176-Si and GmMYB176-OE roots by qPCR analysis are consistent with the previous work (Yi et al., 2010) but these results did not match with the RNAseq data. The expression level of *GmCHI1B2* was 3.6 times lower in GmMYB176-OE than control (Table 2). However, no significant difference in its expression level was detected in GmMYB176-Si tissues compared to control by qPCR analysis (Figure 4).

### Alteration of *GmMYB176* Transcript Level Affects Metabolite Production in Soybean Hairy Roots

**FIGURE 5:**
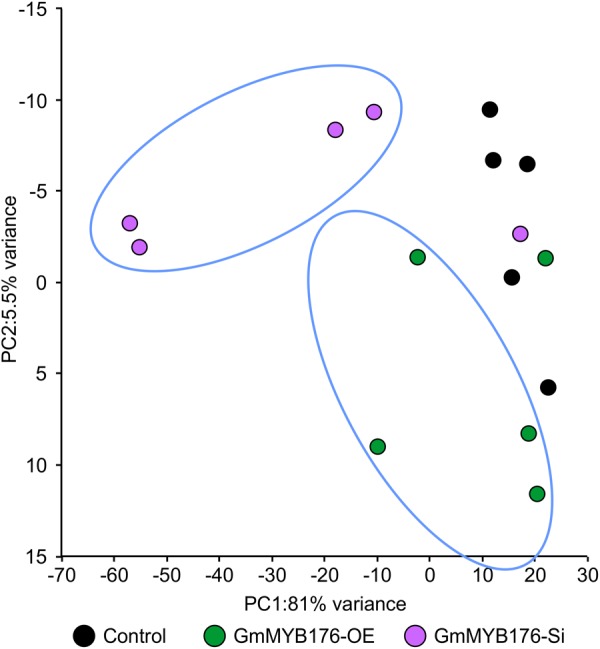
Principal component analysis (PCA) of metabolite profiles in soybean hairy roots with altered *GmMYB176* expression. Dots represent replicates. Five biological replicates for each of the samples: GmMYB176-Si, GmMYB176-OE, and controls were used for the metabolite analysis in positive mode.

**FIGURE 6:**
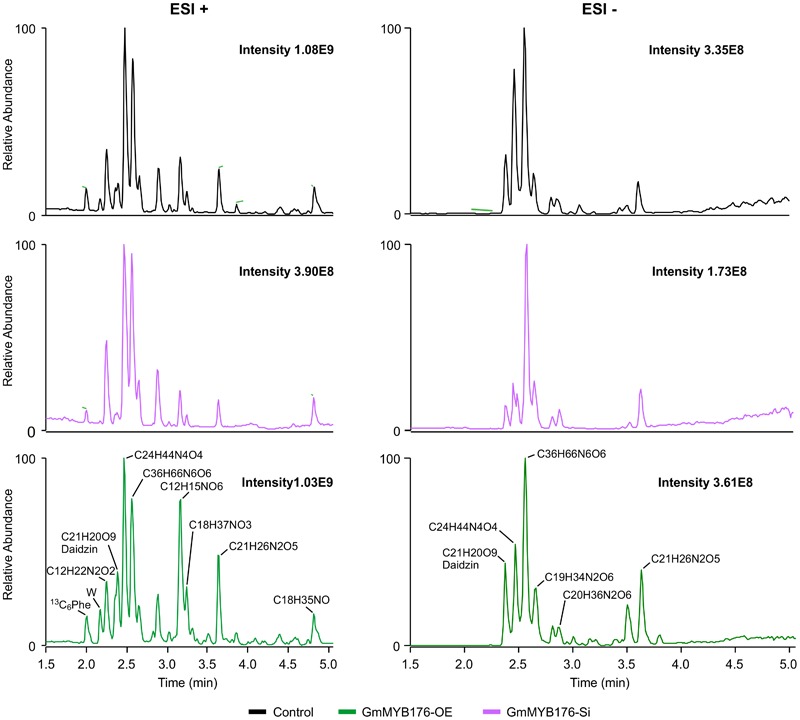
LC-MS chromatograms of soybean hairy roots. Relative abundance of metabolites in GmMYB176-Si, GmMYB176-OE and control samples are shown. Black, red, and green peaks represent control, GmMYB176-Si, and GmMYB176-OE, respectively.

**FIGURE 7:**
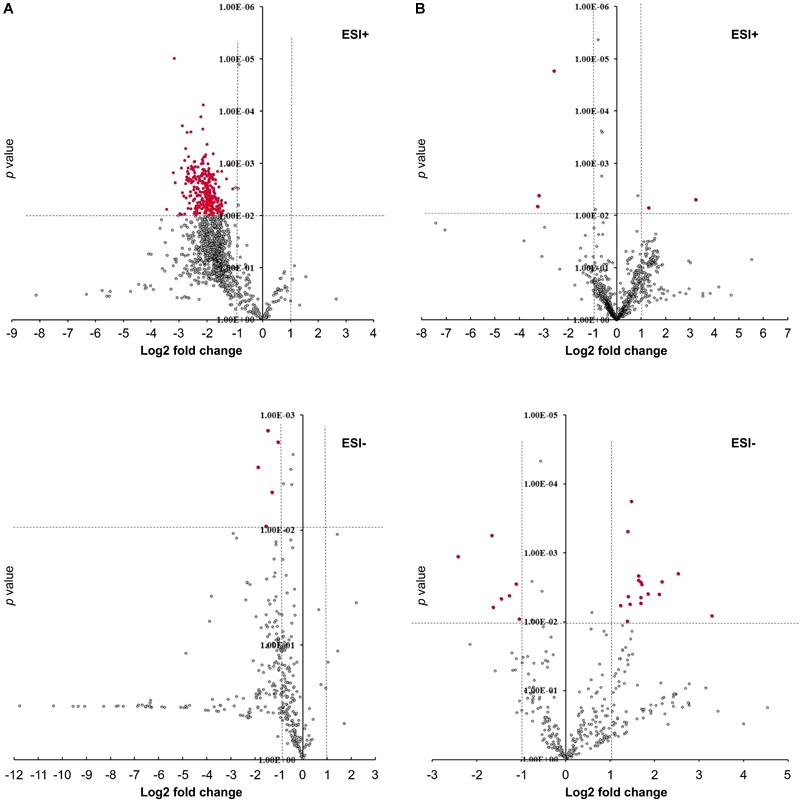
Volcano plots showing differentially produced metabolite features in hairy roots. **(A)** GmMYB176-Si vs. control, **(B)** GmMYB176-OE vs. control. Red dots represent metabolite features that were more than 2-fold differentially produced at *P* < 0.01. Both +ve and –ve modes are shown.

To determine the effect of GmMYB176 on overall metabolite production in soybean roots, we performed a metabolite profile analysis of transgenic roots from GmMYB176-OE and GmMYB176-Si samples, and compared those with the control hairy roots. The PCA plot of metabolomics data indicated a metabolic shift when *GmMYB176* transcript levels were altered (Figure 5). Additionally, out of 5 replicates for each sample set included in the study, one replicate from each of the three sets were outliers in the PCA plot. These outliers were also included in the analysis of total metabolite changes since they show the heterogeneity of these biological samples. Overall, it was evident that silencing of GmMYB176 had a greater effect on the metabolite production than overexpression (Figure 5). Figure 6 displays metabolic profiles demonstrating the impact of *GmMYB176* silencing and overexpression on total metabolites in the hairyroot samples. Several metabolites were produced either at higher level or lower level in GmMYB176 altered samples compared to control. A total of 995 metabolite features (in both HESI+ and HESI-) detected by XCMS in GmMYB176-Si and 149 in GmMYB176-OE tissues were differentially produced compared to control (Figure 7 and Supplementary Table S4). An increase in the levels of 121 metabolite features and decrease in the level of 28 metabolite features was observed in GmMYB176-OE samples compared to control while an increase in the levels of only 3 and decrease in 992 metabolites was found in GmMYB176-Si samples compared to control (Supplementary Table S4, Figure 7 and Supplementary Figure S6). The common contaminants from LC-MS/MS were removed from the list of metabolites in Table S4 according to the procedure described by Keller et al. (2008). The number of significantly differentially produced metabolites in GmMYB176-Si tissues was higher than GmMYB176-OE tissues relative to controls and in the positive mode of HESI than in the negative mode of HESI.

### A Targeted Approach to Identify Differentially Produced Isoflavonoids

**FIGURE 8:**
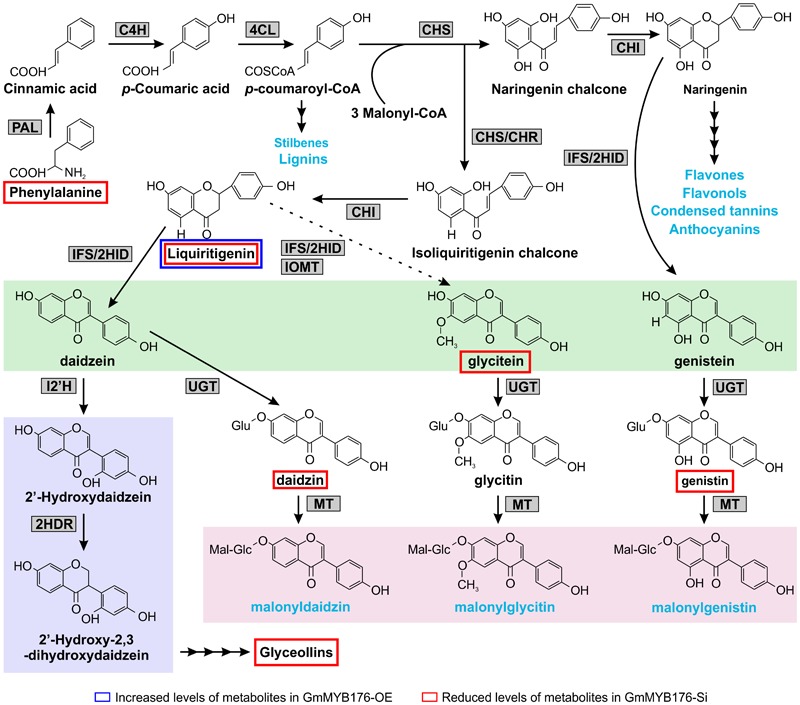
Differentially accumulated isoflavonoids in GmMYB176-Si and GmMYB176-OE hairy roots compared to controls. The multiple *arrows* indicate multiple steps in the pathway and the *dotted arrow* indicates speculated steps. The major metabolites synthesized from the phenylpropanoid pathway are shown in *blue* text. The three isoflavonesaglycones, daidzein, glycitein, and genistein are highlighted in *green* and malonylisoflavones are highlighted in *pink*. The induced isoflavonoid phytoalexin synthesis branch is highlighted in purple. Enzyme abbreviations are mentioned in Figure 1 [Modified from Anguraj Vadivel et al. (2015)].

Our metabolomics study identified a total of 1144 metabolite features that were differentially produced either in GmMYB176-Si or GmMYB176-OE samples compared to controls indicating massive metabolomic implications with the alteration of GmMYB176 expression (Supplementary Table S4). To identify differentially produced isoflavonoids and its precursor metabolites that were affected by the alteration of *GmMYB176* expression in soybean hairy roots, we used accurate *m/z* (< 3.0 ppm) and compared their retention time with the authentic standards (Table 3). Interestingly, silencing of GmMYB176 affected the entire pathway, and reduced the level of phenylalanine, the first precursor amino acid, to the phytoalexin glyceollin (Figure 8 and Table 3). Several other isoflavone aglycones, isoflavone glycosides and liquirigenin were also found at lower level in GmMYB176-Si compared to control roots. The targeted approach identified increased level of only one iso(flavonoid)-specific metabolite liquiritigenin in GmMYB176-OE samples compared to controls. Liquiritigenin level was reduced 5 times in GmMYB176-Si tissues and elevated 2.5-times in GmMYB176-OE tissues compared to the control. Since a standard for glyceollin was not available commercially, its identification was performed based on *m/z* only by comparing fragment ions of the compound as determined by Quadri et al. (2014).

**Table 3:** Differentially produced metabolites identified and confirmed by targeted approach.

*mz*	rt	*P*-value	FC	ESI	Sample (vs. Control)	Metabolite
255.0643	2.39	0.060	0.30	+	GmMYB176-Si	Daidzein (in source fragment)
271.0592	2.54	0.091	0.33	+	GmMYB176-Si	Genistein (in source fragment)
417.1167	2.39	0.074	0.31	+	GmMYB176-Si	Daidzin
433.1117	2.54	0.067	0.32	+	GmMYB176-Si	Genistin
257.0842	2.83	0.084	0.17	+	GmMYB176-Si	Liquiritigenin
285.0750	2.90	0.076	0.29	+	GmMYB176-Si	Glycitein
166.0858	2.02	0.032	0.13	+	GmMYB176-Si	Phenylalanine
339.2882	4.42	0.081	0.21	+	GmMYB176-Si	Glyceollin
255.0699	2.84	0.036	2.43	-	GmMYB176-OE	Liquiritigenin

*mz, mass to charge ratio; rt, retention time; FC, fold change.*

## Discussion

MYB transcription factors coordinate the expression of multiple genes in the phenylpropanoid pathway, thereby affecting the metabolite levels in plants (Bruce et al., 2000; Vom Endt et al., 2002). The coordinated expression of phenylpropanoid genes has been shown to involve largely R2R3 MYBs (Tian et al., 2017). Previously, we demonstrated that an R1 MYB, GmMYB176, regulates *GmCHS8* transcript levels and affects isoflavonoid accumulation in soybean (Yi et al., 2010). Here we present an overview of GmMYB176-regulated synthesis of isoflavonoids by integrating the global transcripts and metabolites affected by the alteration of *GmMYB176* level.

High sample heterogeneity was observed within a hairy root sample set used in the transcriptome analysis as each biological replicate contained pooled transgenic roots (Figure 3). As compared to control hairy roots, a total of 33 DEGs in GmMYB176-Si and 5727 in GmMYB176-OE roots were identified. The soybean genome is predicted to generate 88,647 transcripts. The transcriptome analysis of hairy roots with altered *GmMYB176* levels revealed that 0.037 and 6.46% of the total soybean transcriptome were differentially expressed in GmMYB176-Si and GmMYB176-OE, respectively, in comparison to controls suggesting that GmMYB176 regulates multiple genes involved in various metabolic processes including isoflavonoids.

Using a targeted approach, we identified 9 (iso)flavonoid gene families encoded by 25 gene loci that were differentially expressed in GmMYB176-OE (Table 2). Among the 25 DEGs, 16 were downregulated while 6 were upregulated suggesting that GmMYB176 acts as both positive and negative regulators of (iso)flavonoid genes. This dual role of GmMYB176 was also observed for the members of the same family. For example, out of fourteen *GmCHS* genes (Anguraj Vadivel et al., 2018), three *GmCHS*s [Glyma.08G109400 (*GmCHS1*), Glyma.08G109300 (*GmCHS3a*), and Glyma.08G109500 (*GmCHS9*)] are upregulated while four [Glyma.09G075200 (*GmCHS6*), Glyma.11G011500 (*GmCHS8*), Glyma.02G130400 (*GmCHS10*), Glyma.01G091400 (*GmCHS11*)] are downregulated upon overexpression of GmMYB176. Since genes belonging to the same family contain high sequence identity among each other, it is unknown how accurately the sequence reads were assigned to a specific gene during the analysis. Nonetheless, the genes present on the same chromosome and locations closer to each other (*GmCHS1, GmCHS3a*, and *GmCHS9*) showed similar expression pattern providing confidence in the analysis. The key isoflavonoid gene *GmIFS* (Jung et al., 2000) was also downregulated in GmMYB176-OE. No known glycosyltransferase (Dhaubhadel et al., 2008; Di et al., 2015; Rojas Rodas et al., 2016) were found differentially expressed in GmMYB176-OE or GmMYB176-Si compared to control.

Despite the fact that RNAseq is a powerful tool used in the modern biological studies, there are some limitations to the technique when using it in the organisms with complex genomes. The expression profiles of 6 isoflavonoid genes (*GmIFS1, GmCHS1, GmCHS6, GmCHS9*, and *GmPT10a*) tested by qPCR were consistent with those analyzed by RNA-seq (Figure 4 and Table 2) while the expression levels of remaining 6 genes were not. The reasons for this discrepancy are due to the differences in genome structures and overlapping gene models, genes encoding short transcripts, as well as gene family size (Hirsch et al., 2015). Since gene-specific primers were used to amplify the unique gene products and the amplicons were sequence confirmed, qPCR results can be reliable in these cases. Similar conundrums were observed in a recent study where transcriptome analysis was performed of rice plants attacked by rice stem borer (Liu et al., 2016).

Down regulation of a large number of genes in GmMYB176-OE suggests that GmMYB176 could be a transcriptional repressor. Soybean contains 329 transcriptional regulators with repressor motif EAR (LXLXL) (Kagale and Rozwadowski, 2010). Even though GmMYB176 contains an EAR motif in the *C*-terminal, transcriptional activation of *GmCHS8* by GmMYB176 has been demonstrated in soybean embryo protoplast (Yi et al., 2010). Post-translational modifications including phosphorylation could alter functions of EAR motif containing proteins (Kagale et al., 2010; Kagale and Rozwadowski, 2011). The effect of phosphorylation of a transcription factor with EAR motif on its function has been reported in Arabidopsis, where overexpression of ERF8 with EAR motif acts both as transcriptional activator and repressor (Cao et al., 2018). Similarly, phosphorylation of a EAR-motif containing transcriptional repressor (ARF2) abolishes its repressor activity in Arabidopsis (Zhao et al., 2016). Therefore, phosphorylation state of GmMYB176 and presence or absence of co-repressors/co-activators may affect the target gene expression. Additionally, it is possible that its interacting partner may influence mechanism of gene regulation by GmMYB176 which requires further investigation.

Plants synthesize specialized metabolites that define the biochemical phenotype of a cell or tissue and can be viewed as the end products of gene expression (Sumner et al., 2003). Using a non-targeted approach we identified 995 and 149 differentially produced metabolite features in GmMYB176-Si and GmMYB176-OE tissues, respectively. Many of these metabolite features corresponded to formula which contained nitrogens and were likely not directly linked to the phenylpropanoid pathway (Supplementary Table S4). This massive alteration in metabolite production stretching beyond the phenylpropanoid pathway suggests that GmMYB176 has direct or indirect implications to multiple pathways. To address the specific objectives of this study, we used a targeted approach to identify differentially produced isoflavonoids in GmMYB176-Si and GmMYB176-OE samples. Phenylalanine is the first substrate that is utilized into the phenylpropanoid biosynthesis (Figure 1). A small change in phenylalanine level could affect the production of many downstream metabolites. The Phenylalanine level was 5 times lower in GmMYB176-Si tissues relative to the control (Table 3) which may result in reduced production of downstream metabolites (Figure 8). However, no difference in the levels of cinnamic acid or other immediate downstream metabolites was observed with the alteration of GmMYB176 level. Even though differences in the levels of transcript accumulation of pathway enzymes such as GmCHSs, GmCHR, and GmCHIs were noticed by gene expression analysis, it did not show any effect on the immediate product formation. It is possible that this pathway is extremely active and the intermediate metabolites (except for liquiritigenin) are present only transiently before they are used as a substrate for the successive reaction. This speculation can be explained by our findings that only phenylalanine, the starting molecule, and the isoflavones and glyceollins were found in our targeted metabolite analysis (Table 3 and Figure 8). Phytoalexin glyceollin production is the final step in isoflavonoid pathway (Welle and Grisebach, 1988). Furthermore, overexpression of GmMYB176 directs the flux toward isoflavonoid biosynthesis by downregulating other phenylpropanoids such as flavonoids and lignans (Supplmentary Figures S4, S5). A maize R2R3 transcription factor, ZmMYB31, indirectly regulates anthocyanin production by repressing lignin biosynthesis and redirect the metabolic flux to the flavonoid biosynthetic pathway (Fornalé et al., 2010).

Taken together, GmMYB176 regulates the accumulation of at least six metabolites in the isoflavonoid biosynthetic pathway in soybean roots. Furthermore, it regulates multiple genes and metabolite production in soybean. There was not much correlation between transcriptomics and metabolomics analyses performed in the same sample sets which illuminate the complexity of GmMYB176-mediated gene regulation. It appears that coordinated expression of pathway genes, substrate flux, product threshold level contribute to dynamic of isoflavonoid pathway regulation.

## Author Contributions

AKAV performed the experiments and analyzed the data and wrote the draft manuscript. SK analyzed RNAseq data and JR performed metabolite data analysis. SD conceived and designed the experiments, supervised all aspects of the project and prepared final draft manuscript. All the authors read and approved the article.

## Conflict of Interest Statement

The authors declare that the research was conducted in the absence of any commercial or financial relationships that could be construed as a potential conflict of interest.

## Footnotes

1 https://www.rdocumentation.org/packages/DESeq2/versions/1.12.3/topics/rlog

2 http://phytozome.jgi.doe.gov/phytomine/

3 www.plantcyc.org

4 https://phytozome.jgi.doe.gov/phytomine/begin.do
