# Supplementary material for: GmMYB176 Regulates Multiple Steps in Isoflavonoid Biosynthesis in Soybean
Source: Front Plant Sci. 2019 May 3;10:562. doi: 10.3389/fpls.2019.00562 (PMC6509752; doi:10.3389/fpls.2019.00562)
Supplement: Supplementary file 1 [file Table_1.DOCX]

Table S1. Sequence of oligonucleotides used for qPCR

| **Gene** | **Primer name** | **Sequence (5' to 3')** | **Amplicon size (bp)** |
| --- | --- | --- | --- |
| *GmMYB176* | MYB176F | GAACAGGTCGAGAATCAAGACA | 103 |
|  | MYB176R | GATACACTGGCATCGCTGGAAA |  |
| *GmCHS8* | CHS8/qRTf1 | GCTCCCATTTAATTGATTTCTGAA | 245 |
|  | CHS78/2EXNr | GACTTGTCACACATGCGCTGGAA |  |
| *GmIFS1* | IFS1qF | AAACTAGTGTCTTGCCATCGC | 158 |
|  | IFS1qR | GGGCCGGGAAAAAAAAATTGTC |  |
| *Gm20C4H* | qGm20C4H-F | ACTACTCTGCCCCCAGGTCC | 168 |
|  | qGm20C4H-R | AAGCTCGGGGTCAGAGACCA |  |
| *GmPT10a* | PT10a-QF1 | AATCGCTTCATCGTGGACGG | 157 |
|  | PT10a-QR1 | TGGCCTCTTCAACACAACGG |  |
| *CON4* | CON4F | GATCAGCAATTATGCACAACG | 106 |
|  | CON4R | CCGCCACCATTCAGATTATGT |  |
| *GmCHS9* | qCHS9-F | CACGCGTCTACCTTAACGGG | 165 |
|  | qCHS9-R | GCTAGCTTGCACCAAAGAATGA |  |
| *GmCHS6* | qCHS6-F | CTGTTGTGCTTCGCAGTGTC | 112 |
|  | qCHS6-R | AAGCATATAGAAAGACGGGAAA |  |
| *GmCHS11* | qCHS11-F | TGCAAAGTGAATAGCTTTTGTCCA | 100 |
|  | qCHS11-R | CCTTGTTCACAATTTCACAAAGTCC |  |
| *GmCHS1* | qCHS1-F | ACTGTCTCGAACCAGGTTTACT | 192 |
|  | qCHS1-R | CGCTGTCCATACTTTTGTGTCA |  |
| *GmCHS10* | qCHS10-F | TTCCTTCTCTTTTGCTTCATTTTGT | 155 |
|  | qCHS10-R | AGTGAGCCACAAAGAATGTTTTA |  |
| *GmCHI1B2* | 1B2F | CCACTTTGTTTGAATTTGTACCCC | 214 |
|  | 1B2R | TTCCTATTTATGTGCTTTTACCTATAACATG |  |
| *GmCHS13* | qCHS13-F | CCCTCGGAAATACCAGGGAA | 169 |
|  | qCHS13-R | CGAAAACGAAATGCAATGCCG |  |
