# Supplementary figures and images for: GmMYB176 Regulates Multiple Steps in Isoflavonoid Biosynthesis in Soybean
Source: Front Plant Sci. 2019 May 3;10:562. doi: 10.3389/fpls.2019.00562 (PMC6509752; doi:10.3389/fpls.2019.00562)

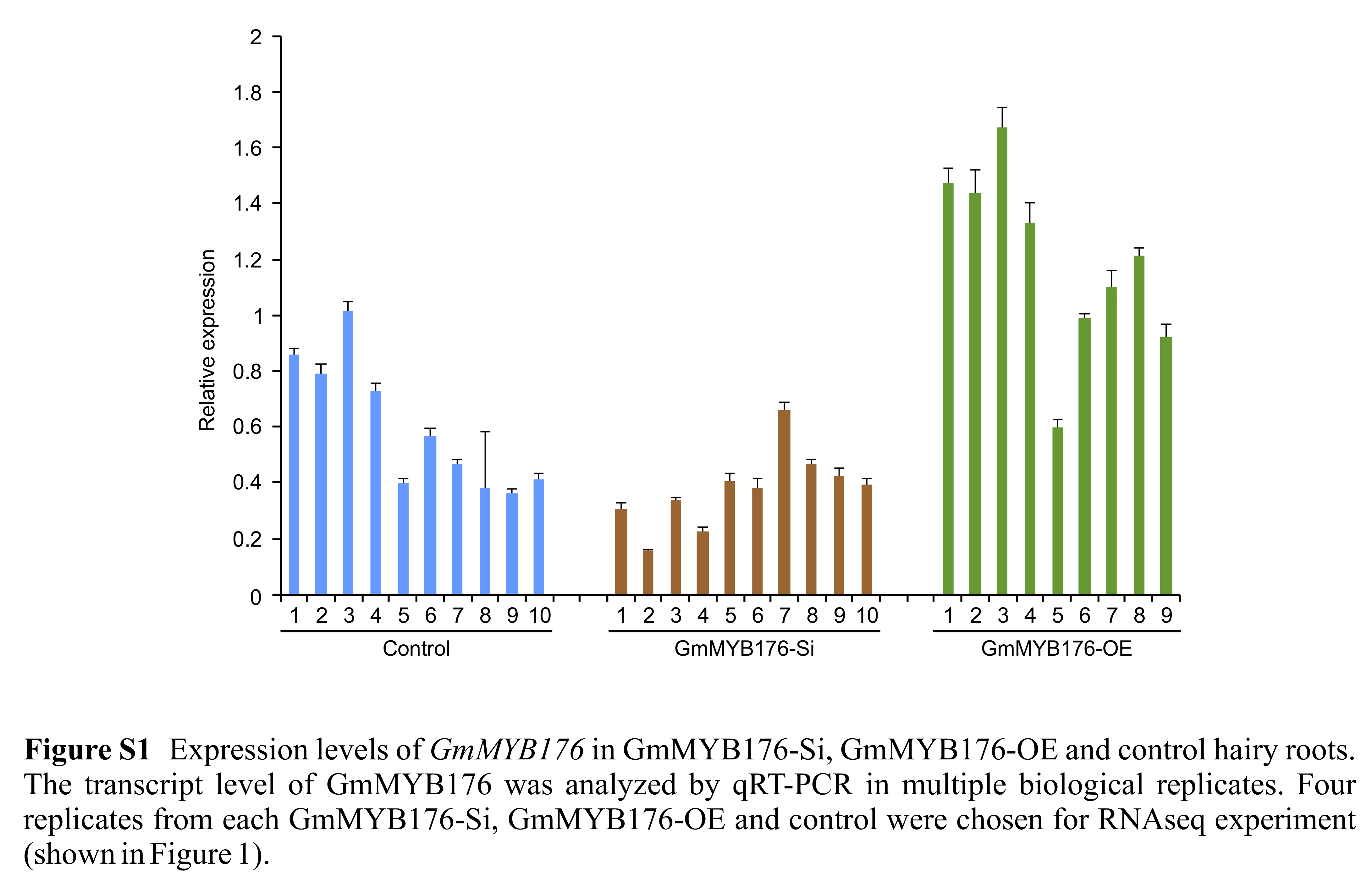

Supplement: Supplementary file 5 [file Image_1.TIF]

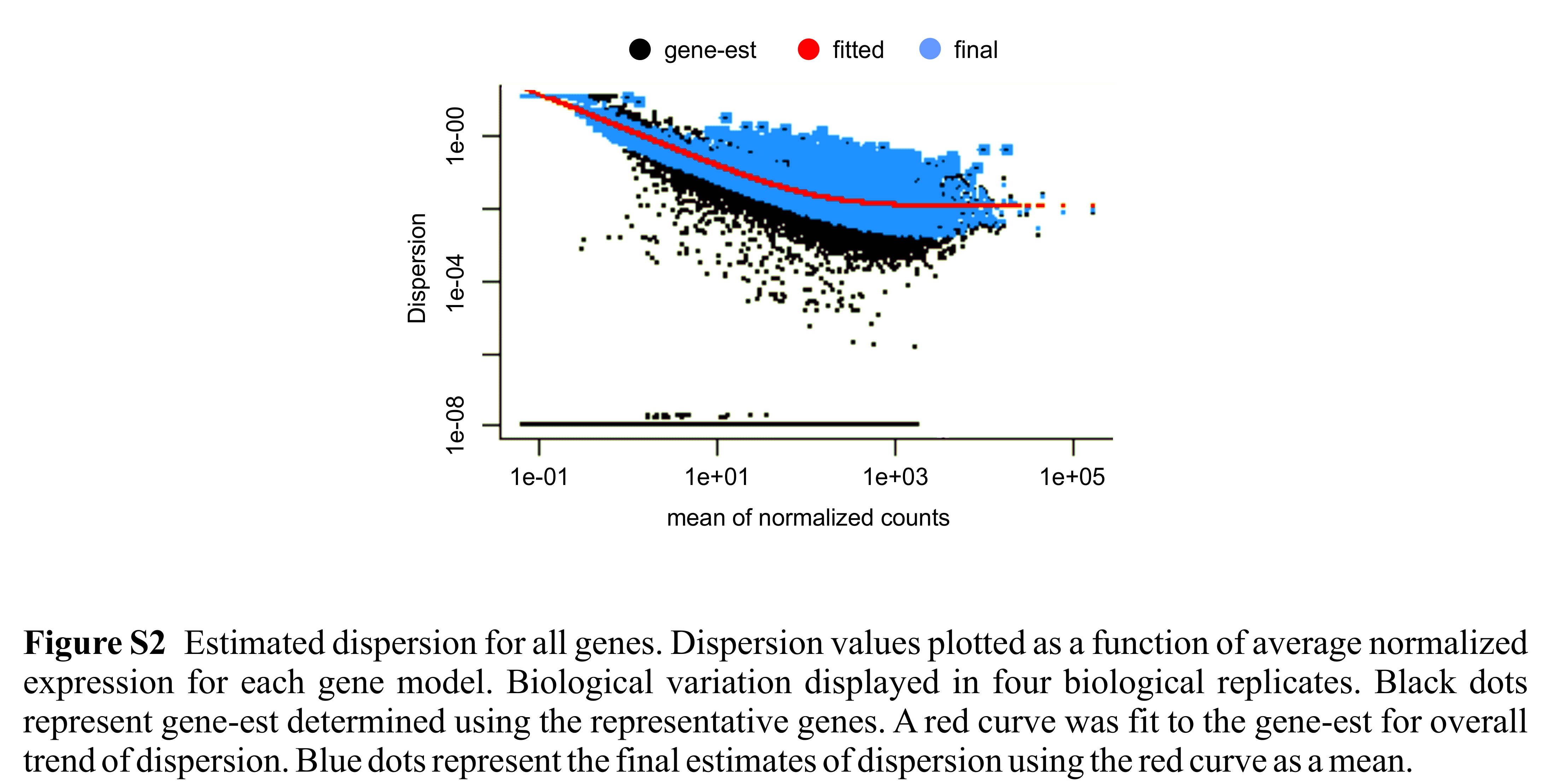

Supplement: Supplementary file 6 [file Image_2.TIF]

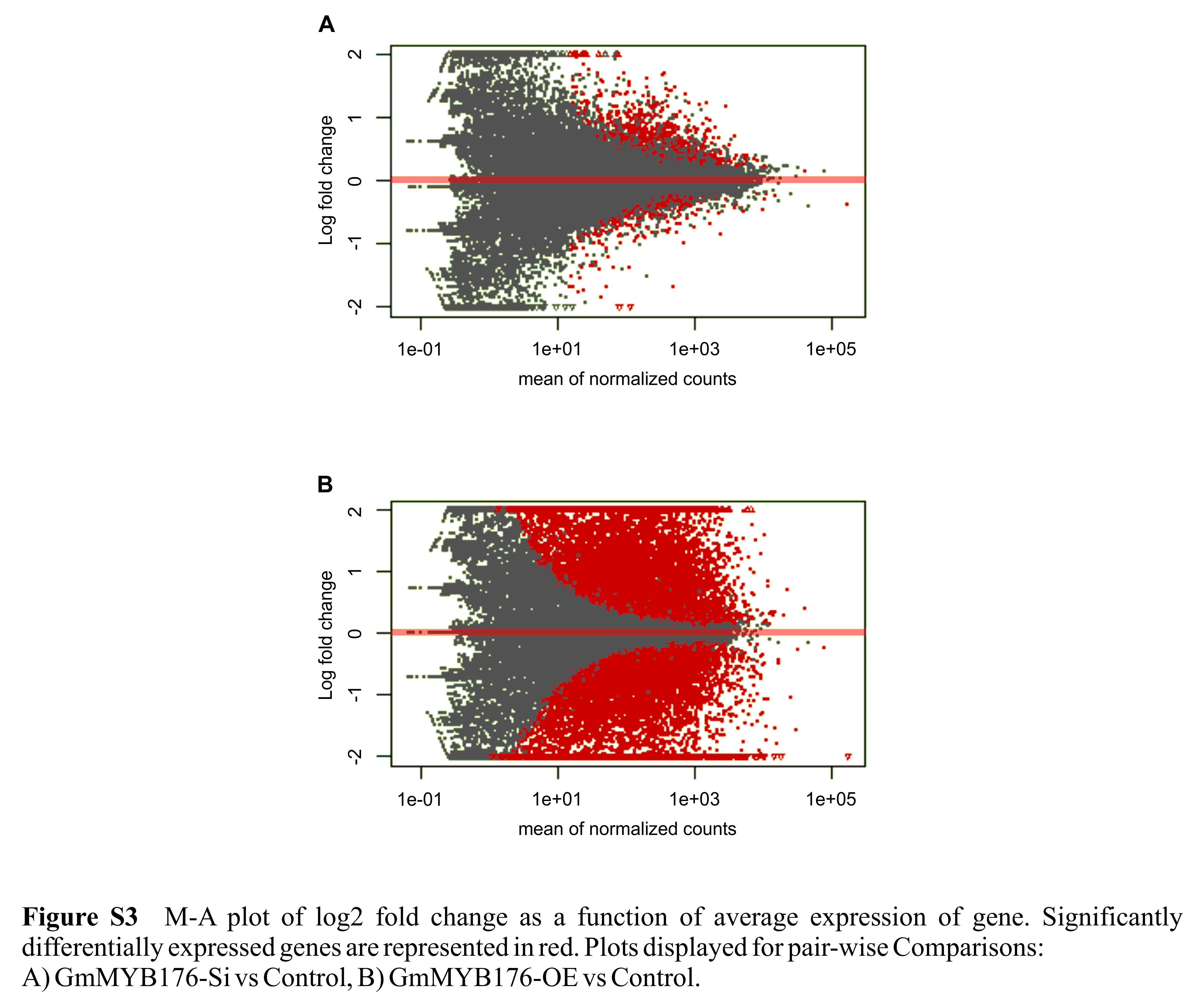

Supplement: Supplementary file 7 [file Image_3.TIF]

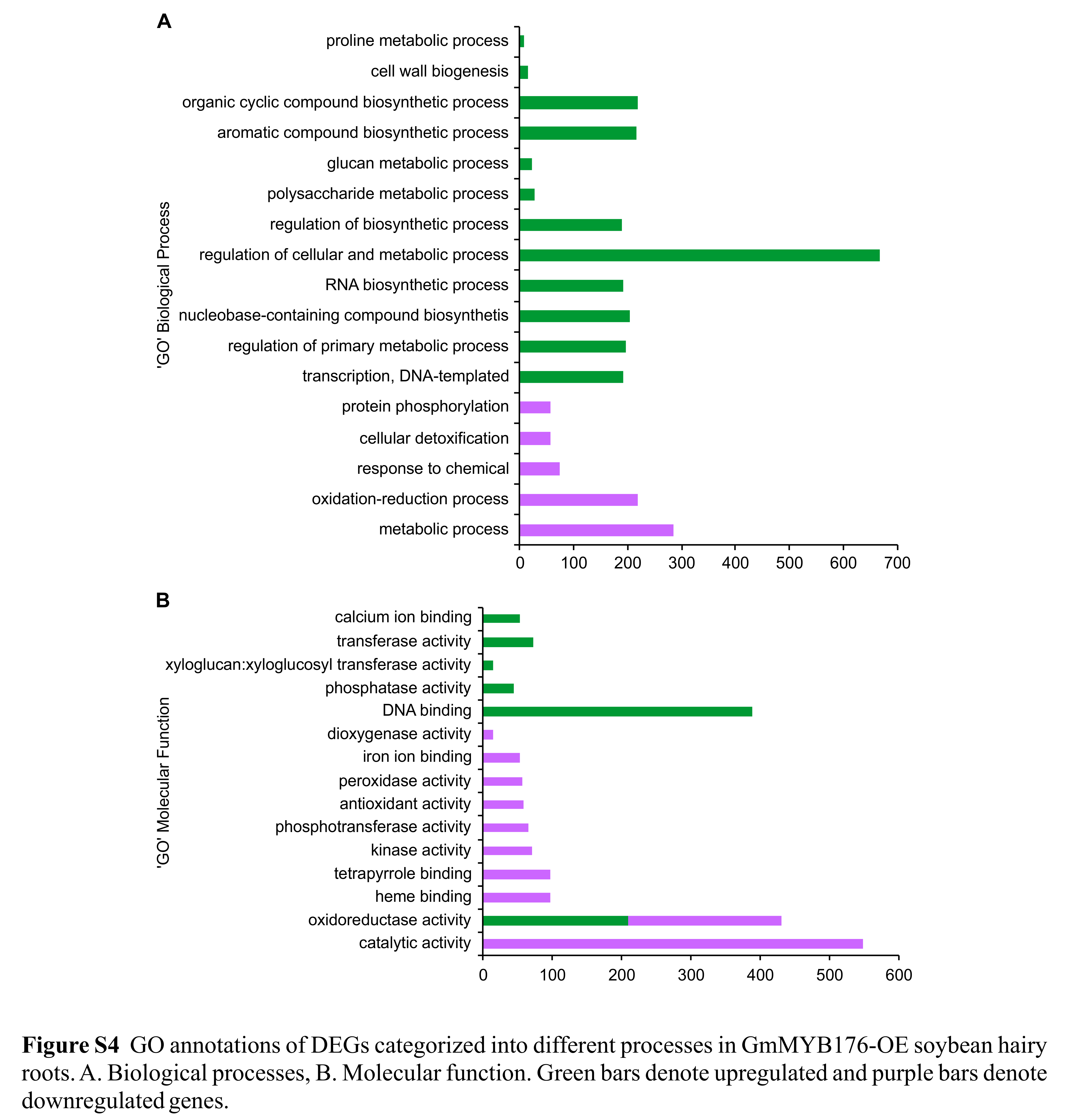

Supplement: Supplementary file 8 [file Image_4.TIF]

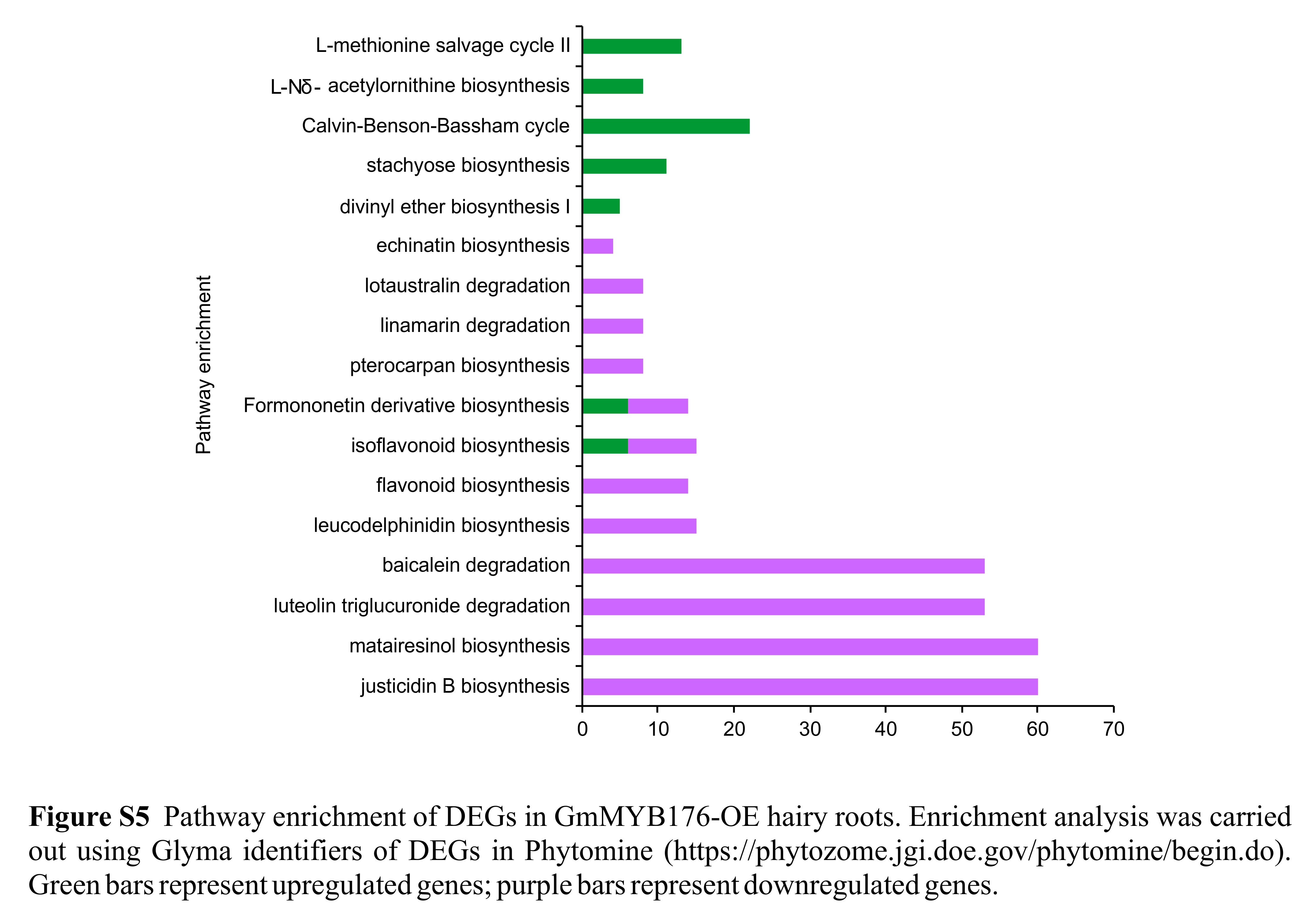

Supplement: Supplementary file 9 [file Image_5.TIF]

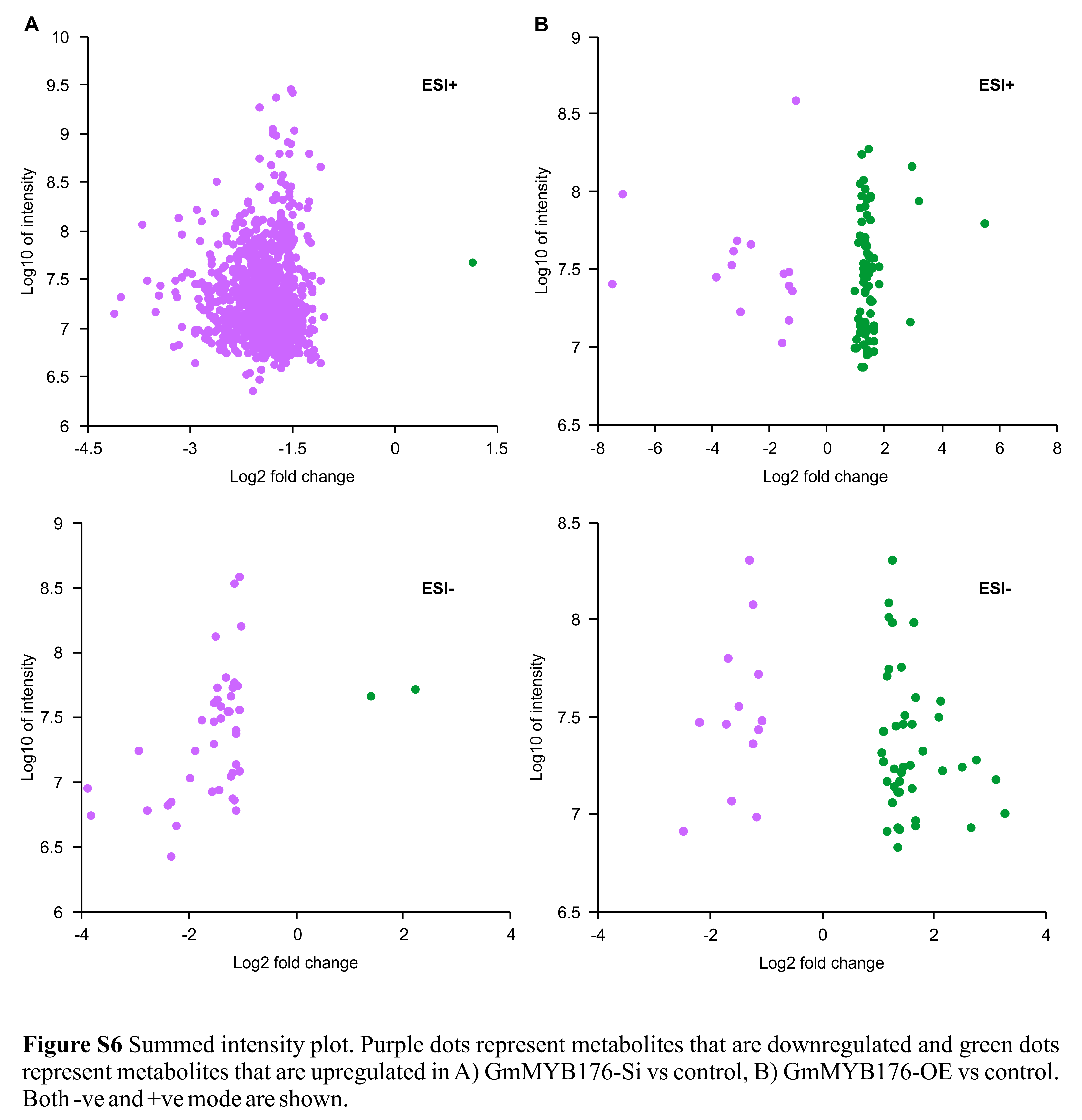

Supplement: Supplementary file 10 [file Image_6.TIF]
